# Supplementary material for: Impact of Developing Dialysis-Requiring Acute Kidney Injury on Long-Term Mortality in Cancer Patients with Septic Shock
Source: Cancers (Basel). 2023 Jul 14;15(14):3619. doi: 10.3390/cancers15143619 (PMC10377237; doi:10.3390/cancers15143619)

Table S1. Characteristics of the study population according to 1-month mortality

Characteristics	Total (n = 42477)	1-month survivors (n = 20412)	1-month non-survivors (n = 22065)	*P*-value
Dialysis	5449 (12.8)	2113 (10.4)	3336 (15.1)	< 0.001
Female	15058 (35.5)	7594 (37.2)	7464 (33.8)	< 0.001
Age (year)*	69.0 (59.0 – 76.0)	68.0 (58.0 – 76.0)	69.0 (60.0 – 77.0)	< 0.001
Insurance	39185 (92.3)	18708 (91.7)	10477 (92.8)	< 0.001
CCI* (continuous)	6.0 (3.0 – 9.0)	5.0 (3.0 – 9.0)	7.0 (4.0 – 10.0)	< 0.001
Underlying disease				
Hypertension	22338 (52.6)	10466 (51.3)	11872 (53.8)	< 0.001
Diabetes mellitus	16321 (38.4)	7546 (37.0)	8775 (39.8)	< 0.001
Congestive heart failure	5475 (12.9)	2433 (11.9)	3042 (13.8)	< 0.001
COPD	4903 (11.5)	2040 (10.0)	2863 (13.0)	< 0.001
Liver cirrhosis	4823 (11.4)	1842 (9.0)	2981 (13.5)	< 0.001
Cancer type				
Solid**	39811 (93.7)	19179 (94.0)	20632 (93.5)	< 0.001
Hematologic***	2666 (6.3)	1233 (6.0)	1433 (6.5)	< 0.001

***Data presented as median (interquartile range).**

**Solid cancer included brain, lung, liver, colon, stomach, gall bladder, pancreas, and lymphoma.

***Hematologic cancer included multiple myeloma and leukemia.

Abbreviations: CCI = Charlson Comorbidity Index; COPD = chronic obstructive pulmonary disease.

Table S2. Univariate and multivariate Cox-proportional hazard analysis for predicting 1-month mortality

Variables	Univariable			Multivariable		
	HR	95% CI	*P*	Adjusted HR	95% CI	*P*
Dialysis	1.295	1.248 – 1.344	< 0.001	1.338	1.288 – 1.389	< 0.001
Female	0.907	0.882 – 0.933	< 0.001	0.946	0.920 – 0.973	< 0.001
Age	1.007	1.006 – 1.008	< 0.001	1.010	1.009 – 1.011	< 0.001
Hypertension	1.073	1.044 – 1.101	< 0.001	0.948	0.921 – 0.977	< 0.001
Diabetes	1.079	1.050 – 1.109	< 0.001	0.884	0.859 – 0.911	< 0.001
CHF	1.122	1.080 – 1.165	< 0.001	0.999	0.959 – 1.040	0.955
COPD	1.216	1.170 – 1.265	< 0.001	1.094	1.050 – 1.139	< 0.001
LC	1.372	1.320 – 1.426	< 0.001	1.335	1.283 – 1.389	< 0.001
CCI	1.054	1.051 – 1.058	< 0.001	1.061	1.057 – 1.065	< 0.001
Solid cancer*	Reference			Reference		
Hematologic cancer**	1.025	0.971 – 1.081	0.373	1.214	1.149 – 1.283	< 0.001

*Solid cancer included brain, lung, liver, colon, stomach, gall bladder, pancreas, and lymphoma.

**Hematologic cancer included multiple myeloma and leukemia.

Abbreviations: HR, hazard ratio; CI, confidence interval; CHF = congestive heart failure; CKD = chronic kidney disease; LC = liver cirrhosis; CCI = Charlson Comorbidity Index.

Figure S1. Kaplan-Myer survival curves of the 1-month mortality

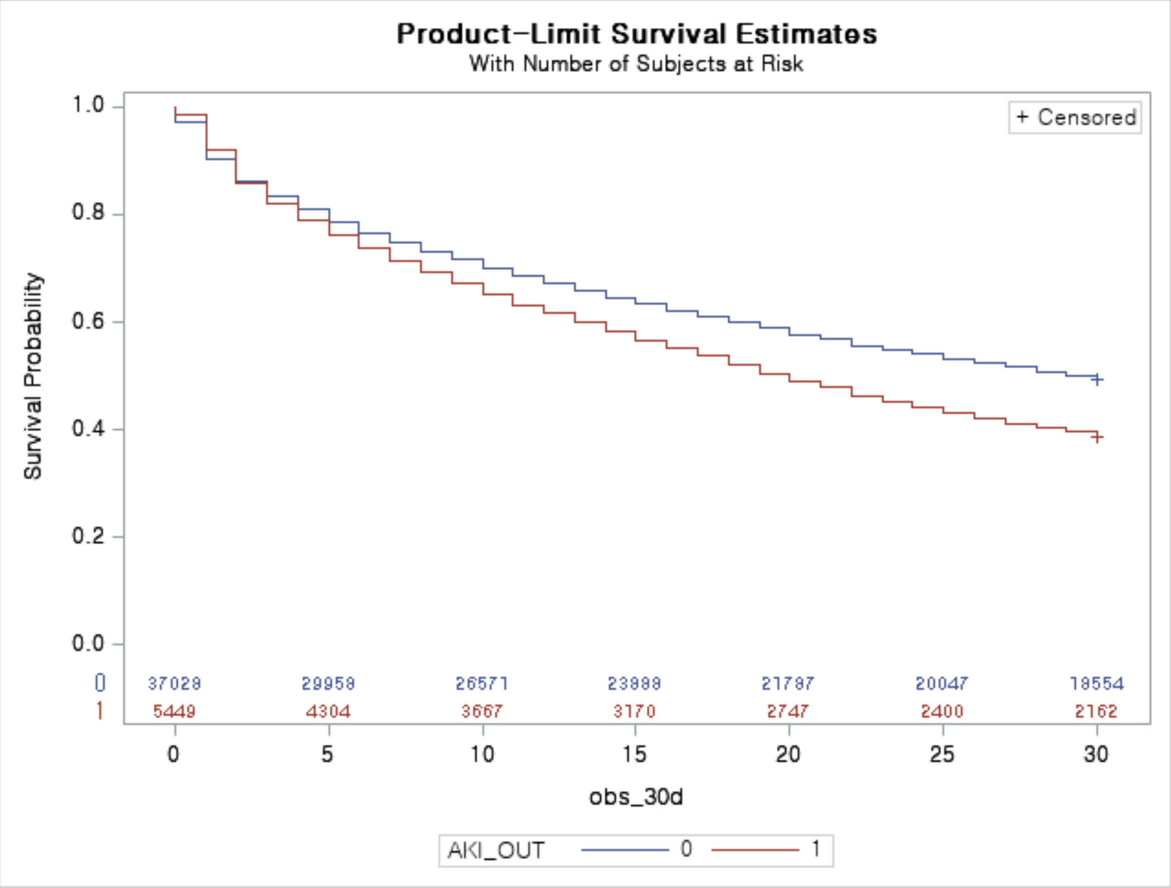

Supplement: Supplementary file 1 [file cancers-15-03619-s001.zip › cancers-2454317-supplementary.pdf]
